# The Formation Mechanism of Impulse Buying in Short Video Scenario: Perspectives From Presence and Customer Inspiration

**DOI:** 10.3389/fpsyg.2022.870635

**Published:** 2022-06-28

**Authors:** Peng Gao, Yuanyuan Zeng, Yu Cheng

**Affiliations:** ^1^Department of Information Management and Information System, School of Economics and Management, Northwest University, Xi'an, China; ^2^College English Department, School of Foreign Languages, Northwest University, Xi'an, China

**Keywords:** short video, presence, customer inspiration, impulse purchase, social factors

## Abstract

It has been found in many cases that consumers are prone to exhibit impulsive buying behavior that is manifested as being immediate, emotional, and irresponsible especially under short video scenario. Supported by the customer inspiration theory, this study explores the psychological mechanism underlying impulse purchase in short videos that differentiates the traditional web shopping by the strong sense of presence in short video marketing. On the basis of a questionnaire survey and three laboratory experiments, this study examines the relationship among presence, customer inspiration, and impulse purchase intention. The empirical results point to the fact that social presence, co-presence, and physical presence have significant positive effects on impulse purchase intention, and customer inspiration mediates the effect of social presence, physical presence, and co-presence on impulse purchase intention. Furthermore, it is indicated that social and co-presence have stronger influences on impulse purchase intention than physical presence, thus proving a stronger effect of social factors on impulse purchase intention than physical factors in short video environment. The research results testify the impact of presence on consumer behavior in the upgrading short video marketing and provide valuable reference for marketing strategies to shorten consumers' decision-making time in short video purchase.

## Introduction

In 2020, when the short video platforms, users, and advertisers were enjoying the visual and audio banquet of short videos, short video marketing was manifesting a rapid and high-quality development. Short videos have been defined as the videos that are played and pushed with high frequency on various new media platforms. Since the length of these short videos usually ranges from seconds to minutes (Bi et al., 2021), they are featured by mobile and short-term leisure viewing, and endowed with unique marketing value compared with traditional video ads. Short videos incorporate social activities such as liking, commenting, and sharing to content communication (Shen, 2018). Additionally, the preference analysis by platforms of short video enables more accurate searches of target users, more intuitive information of products, and consequently a higher marketing efficiency (Sheng, 2021). According to the statistics of China Internet Network Information Center (CNNIC), by the end of December 2020, the number of independent short video users had reached 873 million, accounting for 88.3% of the domestic Internet users. Furthermore, with an average of 2-h daily online time, the online duration of short video platforms had surpassed that of instant messaging and become one of the most favorable platforms at an alarming rate (China Internet Network Information Center). On the other hand, based on the tremendous traffic flow of short videos, the “short video + e-commerce” model has been exhibiting an astonishing capacity in directing and guiding shopping since its conception. It is calculated that 56.2% of the short video users have purchased goods and services through short video platforms, 40.9% hold very positive attitude toward short video advertisements and pay constant attention to short video ads in varying degrees (Ding et al., 2021). The same is true in e-commerce shopping platforms. A typical case occurred when “Taobao second floor” launched a short video program “Thousand and One Nights.” The sales in only one of the short video episodes such as “Chinese Mackerel Dumplings” has risen 150-fold than other graphic web sales by 10 a.m. of the next day after the launch of the program. In most cases, impulse buying accounts for about 80% in all sales; for such an impulsive behavior, it can be catalyzed by short videos that will enable users to make a purchase decision in a few seconds. The purchasing behaviors of the short video users are usually perceived as being immediate, emotional, and irresponsible because most of them do not have any purchase plan or intention, and are therefore categorized as impulsive buyers.

It can be seen that short video is a powerful marketing tool for the brand owners because of its strong promotion capacity and the large base of the short video users who are very likely to be impulsed to buy something while watching short videos. However, we are still unclear about the specific psychological mechanism of impulsive purchase inspired by short videos in spite of the limited literature on the issue (Gao et al., 2020). For example, Guo found that the value and fit of information content in short video e-commerce has a significant positive impact on users' purchase intention through the mediating effect of users' sense of pleasure and arousal (Guo et al., 2021). Shi also verified the positive effect of usefulness and conveniences of short videos on users' purchase intention (Shi et al., 2021). However, factors such as the content integration, usefulness, conveniences are not exclusive to short videos, but featured by all the traditional static graphic methods. The prior literature, therefore, neither explain why the purchase conversion rate of short video products is 20% higher than that of the graphic mode in traditional e-commerce (Dong, 2019) nor explain why impulse buying is so prevalent when consumers are watching short videos. So, what are the direct causes to consumers' instant purchase behavior when they are exposed to an easy and lifelike experience? These issues are of great importance but have so far not received an adequate attention from the academia.

A short video, which differs from the traditional graphic displaying method mainly by its more powerful visual experience, stronger impact forces, and greater sense of immersion, creates a stronger sense of presence, which is defined as users' real perception of the media environment in which an individual may feel completely immersed, and is thus considered as a crucial factor that determines consumers' online shopping decisions (Li and Hua, 2021). Presence in short videos can significantly affect consumers' willingness to follow the commodities by stimulating their perceptions of value and trust (Cai et al., 2020). Therefore, a high-level presence is one of the most important factors to promote consumer impulse purchase in short video scenario. Customer inspiration is introduced as customers' temporary motivational state that facilitates the transition from the reception of a marketing-induced idea to the intrinsic pursuit of a consumption-related goal (Böttger et al., 2017). Such extrinsic marketing stimuli as presence in short video situations are the decisive factors to spark customer inspiration that is conceived as an important antecedent variable arousing customer purchase intention (Dong, 2020), and whose strong motivational state coincides with an overwhelming urge to buy the consumer goods. In this case, the customer inspiration actually works as a channel to transform the external stimulus to the instant buying behaviors. Consequently, this study, based on the customer inspiration theory, aims to explore whether presence will further affect consumers' impulse purchase intention, and the signaling effect of short video customer inspiration between presence and impulse purchase intention.

## Theoretical Background and Hypotheses

### Presence in Short Videos and Impulse Purchase

Hailing from “Tele-presence” in communication studies, the concept of presence mainly refers to an experience remotely perceived in a virtual environment (Bulu, 2012). Scholars classified presence into the following three dimensions: Physical presence, social presence, and co-presence (IJsselsteijn et al., 2000; Bulu, 2012). Physical presence is used to describe an individual's subjective experience of being in an environment when the individual is physically in another. In other words, an individuals' experience in virtual environments is almost the same as in real life (Witmer, 1998). Social presence reflects the degree of salience in the interaction between an individual and others (Parker et al., 1976). In a virtual environment, for example, the individuals perceive the medium of communication and connection to each other and create a sense of “warmth,” especially (Hassanein and Head, 2007). With a focus on the psychological connection of minds, co-presence is defined as a sense of “being together” in a virtual environment where individuals perceive the accessibility, availability, and subjection to one another (Goffman, 1963), namely, perceiving others and feeling others are, respectively, actively perceiving us and being part of the group (Bulu, 2012). This study on presence in the field of marketing mainly focuses on virtual shopping centers, online shopping decisions, network community construction, etc. similar to the study by Song et al. (2007) who found that when the consumers are buying clothes online, the perceived presence will stimulate their imagination, thus producing effect on the sense of pleasure in shopping. Hassanein and Head (2007) also indicated that presence could positively affect consumers' perceived usefulness, shopping pleasure, and customer confidence. However, these studies have been conducted in web shopping environment that is too plain to exhibit stronger properties of socialization and interactivity produced in short videos which, compared with text and graphics, is more conducive for users to perceive goods and exhibitors, and experience the sense of being together with them. In other words, short video is capable of providing a higher level of presence than the traditional graphic marketing.

Impulse buying refers to an unplanned consumption behavior caused by consumers' immediate and sudden impulse (Kollat and Willett, 1967). Research shows that impulse buying is a common behavior that accounts for about 80% of the total sales (Zhao and Cai, 2014). A review of the previous literature has sought to tease out the impact factors of consumers' impulse buying behavior (Table 1), which fall into the following three categories: Marketing incentive, individual characteristics, and consumption situation. Marketing incentive refers to the marketing strategies employed by the distributors to attract consumers (e.g., price cut and product trials). Individual characteristics, on the other hand, is the intrinsic determinants of impulsive buying behavior (e.g., consumer buying tendencies). Consumption situation is described as the environment where the consumption occurs (e.g., whether shopping with others or not). Among the controllable marketing incentives, the offline entity marketing is more applicable to tactile and new product trials. Therefore, new incentives that can motivate consumers' impulsive purchase, other than the price discount, need to be further explored for short videos and other online marketing methods alike.

**Table 1:** Impact factors of impulse purchase.

**Category**	**Impact factor**	**Literature**
Marketing incentive	Tactile sense	Peck and Childers, 2003
	Price discount	Hong and Zhe, 2015
	Commodity price	Xiong, 2017
	New product trial	Liu and Fan, 2020
Individual characteristic	Impulse purchase intention	Beatty and Ferrell, 1998
	Shopping pleasure	Dholakia, 2000
	Self-discrepancy of consumers	Luna-Arocas, 2008
	Income	Abratt and Goodey, 1990
	Age	Rawlings et al., 1995
Consumption situation	Normative evaluation	Rook and Fisher, 1995
	Presence of others in shopping	Luo, 2007
	Face threat	Yang et al., 2014
	Authenticity, entertainment and visibility of online live-streaming	Liu et al., 2020
	Sense of power	Liu, 2021

The previous studies have found that the network marketers are skillful in triggering consumers' sense of “being there” by offering fascinating interactive pictures, imaginative product descriptions, and shopping tips. As a result, a positive attitude toward the commodities is promoted among the consumers who would feel easier to make a purchase decision (Jiang and Benbasat, 2007). Some studies explore the influencing mechanism of “atmosphere cues” in live streaming context on “impulse purchase intention” (Gong et al., 2019), and some other studies focus on impulse purchase behavior based on “situation theory”. In either case, these studies confirmed that a remote perception in virtual environments may result in impulse purchase, namely, presence leads to impulse buying (Sheng and Zhao, 2018). According to Bulu (2012), social presence has a positive impact on customer satisfaction in virtual environments where individuals are more easily connected to each other and feel more comfortable, less isolated, and consequently are more contented in informal conversation. In addition, co-presence and physical presence will positively affect customer satisfaction. Specifically, individuals are more likely to be inspired to feel satisfied with the product when they feel they are being together with others and being part of the group in a same virtual space (Thrash and Elliot, 2003). Numerous findings have indicated that customer satisfaction can significantly enhance purchase intention, which implies that social presence, co-presence, and physical presence might produce rapid stimuli to customer purchase intention. In their research on web shopping, Jiang et al. discovered that impulse purchase can be stimulated by high-level presence, in which physical presence indirectly affects the impulsive buying behavior of web shoppers, while social presence has both direct and indirect effects on such behavior (Sheng and Zhao, 2018). Similarly, based on the theory of social presence, Chen's (2011) study proved that embedding social cues in shopping websites can improve social presence, positively affect customer pleasure and trust, and ultimately promote the formation of purchase intention. Actually, in the context of short video online shopping, the interaction between customers and platforms, anchors and other customers will bring customers an immersive sense of “being there”. The perceived high-level presence may well expect to raise commodity evaluation, reduce perceived risk, and lift trust and pleasure of the customers (Li et al., 2003)—who, therefore, are apt to nurture an immediate and sudden impulse due to these positive perceptions of commodities. On the basis of these findings, we propose Hypotheses H1a–H1c as follows:

H1a: Social presence has a significant positive effect on impulse purchase intention in short video scenario.

H1b: Co-presence has a significant positive effect on impulse purchase intention in short video scenario.

H1c: Physical presence has a significant positive effect on impulse purchase intention in short video scenario.

### Mediating Effect of Short Video Customer Inspiration

#### Short Video Customer Inspiration and Impulse Purchase

As the explosive development of short video constantly catapulting to become a kingpin in web business, there has been a growing attention in customer psychology and behavior in the scenario of short video. The relevant researches addressed the questions concerning publication marketing (Xie, 2014; Chen, 2019), tourism destination marketing (Li J. Y. et al., 2019; Liu and Gu, 2019; Sun and Zhang, 2019), sporting event marketing (Zhou et al., 2018), film marketing (Tian, 2019), etc. Although most of the existing studies explored short video transmission mechanism (Zhou et al., 2018; Liu and Gu, 2019; Sun and Zhang, 2019), business model (Wang and Sun, 2018; Dong and Bu, 2019; Wang and Guo, 2019), and opportunities and dilemmas brought by short video (Chen, 2019; Tian, 2019), few of them focused on consumer decision-making problems at the micro level of consumer psychology. Studies found that at the cognitive level, the perceived value in short video would positively affect purchase intention, with users' attitude and participation displaying a significant mediating effect (Wang and Huang, 2019). Comparatively, other studies in terms of experience and emotion revealed a significant positive effect of brand experience in short video apps on user loyalty and brand resonance. Also, brand resonance plays a partial mediating role in the impact of behavioral experience and a complete mediating role in intellectual experience on user loyalty (Jia and Hu, 2019). Moreover, both interactive and creative behaviors in short video are found to have a significant positive effect on perceived value and loyalty (Dai and Gu, 2017). In addition, user attitude toward payment is verified to be positively affected by the visual perception created by the content characteristics and emotional perception by the frequency and quality of interaction in short video (Li P. F. et al., 2019). Findings also showed that virtual haptics and pleasure experienced in short video have a significant positive effect on consumers' purchase intention (Guo et al., 2019). In spite of the limited literature in the short history of short video marketing, recent researches on the consumption decision-making mechanism in short video scenario pointed to one of the major characteristics of consumption behavior as having more “human” factors such as “experience,” “resonance,” and “interaction” during the purchase journey in short video than in traditional graphic web page.

A recently proposed theory of customer inspiration is the integration of traditional inspiration theory and consumer behavior theory in psychology, which contributes to the study of human factors on state transition from seeking and willing to accept new ideas (in experience, resonance, interaction, etc.) to changing consumption habits (e.g., purchase behavior). Inspiration is conceptualized in classic psychology as a unique state of intrinsic motivation characterized by evocation, transcendence, and motivation in social psychology. Evocation refers to the inspiration triggered by external factors than by the recipient. Transcendence, however, describes recipient's positive sense of self-enhancement. Then, motivation happens on the moment when inspiration induces a state that is close to motivation, where the recipient feels compelled to actualize new ideas (Thrash and Elliot, 2003). Based on the theory of “inspiration” and practice in marketing, Böttger et al. (2017) (Sheng and Zhao, 2018) first introduced the concept of “customer inspiration” that is defined as a temporary motivational state that facilitates a transition from the acceptance of marketing-induced ideas to the pursuit of consumption-related goals. Furthermore, Böttger et al. illustrated a two-states customer inspiration scale that consists of “inspired-by” and “inspired-to” states. “Inspired-by” state is related to customers' reception of marketing-induced concepts (i.e., evocation) and awareness of new possibilities (i.e., transcendence). On the other hand, “inspired-to” state is connected with consumers' intrinsic pursuit of relevant goals (i.e., motivation). In this case, the customers are eager to implement new ideas such as buying or using a new product rather than simply keep the idea in their minds. In other words, the customers will undergo a transition from “being inspired by the idea” to “being inspired to purchase the product” when they are converting acquired purchase inspiration into specific behaviors (Hoffman and Novak, 1996; Thrash and Elliot, 2003). In this study, ‘short video customer inspiration' is the specific manifestation of customer inspiration in short video context.

The concept of customer inspiration is proposed to connect the reception of activated new ideas to the pursuit of consumption-related goals, and is conducive to further research on the effect of short video on online marketing. Specifically, how to stimulate customers' instant impulsive purchasing behavior while forcefully locking users' attention in the current ever-shortening customer journey. Customer inspiration is usually characterized as a mediating role to facilitate transition from extrinsic marketing stimuli to customer intention and behavior. It has been found by many scholars that customer inspiration has a mediating effect between variables such as perceived product innovation, product localization, vitality of ads, societal compact, and purchase intention (Dong, 2020; Sheng et al., 2020; Tang and Tsang, 2020). In addition, customer inspiration plays a significant impact on customer behavior; for example, it can mediate inspired content and engaged behaviors (Izogo and Mpinganjira, 2020).

Besides, many studies have found evidence that inspiration may lead to customer impulse buying. For example, a large-scale offline questionnaire survey done by Böttger et al. (2017) revealed that the product descriptions with high inspirational content often trigger consumer purchase impulse, while the analysis by Thrash and Elliot (2003) from the perspective of behavior indicated that inspiration elicits intrinsic motivation to the implementation of a new idea that will determine the ultimate behavior, namely, the “inspire by” state. Moreover, the study by Chen et al. (2009) proved that the flow experience components of inspiration will increase the number of unplanned purchases. In general, customers obtain new ideas through the product information cues offered by the marketers. In this process, customers may experience transcendence toward a new mental state that may stimulate an impulse to pursue a relevant goal which is quickly transformed into a purchasing behavior.

#### Presence and Customer Inspiration

Applied in the field of marketing, inspiration also supplements some of its features to customer inspiration, such as the evocation of inspiration that is commonly triggered by an external information other than customers' personal experience, common sense, or self-induced need (Dong, 2020). Therefore, customer inspiration in short videos is actually incentivized by the content in videos. On the other hand, some scholars indicated that a new model that immerses consumers in situations (such as exhibition of product operation, visual search at Pinterest, etc.) can help create rich marketing content that might inspire customers, and thus is conceived as important source of customer inspiration (Böttger et al., 2017). Since immersiveness in situation is the manifestation of the concept of “presence” which describes a remote perceptual experience in virtual or real environment (Shen, 2018), presence may be an important source of inspiration for online customers.

Although no direct literature has been found about the relationship between presence and customer inspiration in virtual shopping scenario, other relative researches can be cited as credence to the implication of the study. On the one hand, some studies have confirmed that customer inspiration includes components of flow experience. For example, Schouten et al. (2007) defined flow experience as feelings acquired by total absorption in an activity, with features such as high concentration, loss of self-awareness, and intrinsic pleasure. They further pointed out that the transcendent customer experience is characterized by emotional intensity, epiphany, singularity, and newness of experience, etc. Therefore, the flow experience can be regarded as a manifestation of inspiration. On the other hand, relevant studies have found that presence in the virtual environment will generate flow experience. The stronger the physical presence and social presence generated by customers, the more likely the customer is to receive the perceptual stimulation in the virtual scene, and the more possible the flow experience is induced (Novak et al., 2000; Schouten et al., 2007; Animesh et al., 2011). In addition, Kim et al.'s (2013) work indicated that a higher degree of co-presence will result in a sense by consumers that they are psychologically closer to their shopping partners, their communication is warmer and friendlier; therefore, their social needs and hedonic value will be satisfied. A high level of hedonic value will deepen the sense of immersion and state of inspiration in terms of concentration. In a study on the relationship between inspirational flow experience and consumption purchase, Chen et al. (2009) found that the consumers' remote perception (presence) affects their flow experience in a significant way. Similarly, an empirical research conducted by Dai and Liu (2015) showed that the social presence of WeChat users has a significant positive impact on flow experience. In summary, short video can effectively inspire the customers by creating a rich marketing content. Specifically, by a higher level of presence, the consumers are more likely to be engaged in the virtual scenario and have a real life-like experience by staying and communicating with network anchors. Consequently, there is greater possibility that consumers will be immersed in short videos and acquire customer inspiration that can ultimately promote the impulse purchase. Accordingly, we propose Hypotheses H2a–H2c as follows:

H2a: Customer inspiration plays a mediating role between social presence and impulse purchase intention in short video scenario.

H2b: Customer inspiration plays a mediating role between co-presence and impulse purchase intention in short video scenario.

H2c: Customer inspiration plays a mediating role between physical presence and impulse purchase intention in short video scenario.

**Figure 1:**
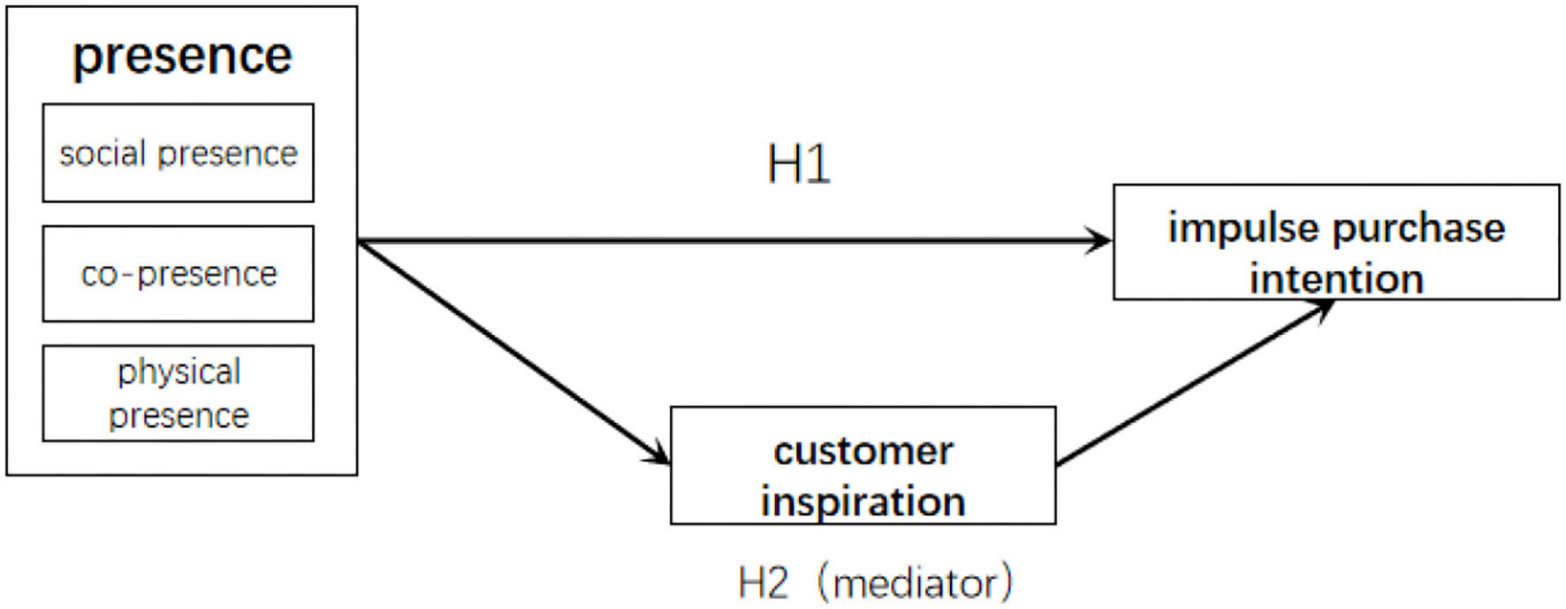
Conceptual model.

In accordance with the formulated hypotheses, a conceptual model is constructed in this study to show the relationship among presence, short video customer inspiration, and impulse purchase intention (Figure 1).

## Study 1: Questionnaire Survey on Presence

### Data Collection

#### Research Samples and Data Sources

This study presents the results of a quantitative analysis of the data collected in a questionnaire survey. The first question is “Have you ever bought something after viewing a short video?” If the response is “No,” the respondent can stop his/her survey; but if it is “Yes,” the follow-up questions will be answered. In the introduction of the questionnaire, the respondent is prompted to “recall an impressive short video ads and answer the questions”; and at the end of the questionnaire, the respondent needs to answer whether he/she “has bought the product promoted in the short video.” Through this part of survey, experienced short video consumers can be selected as the research subject in this study. Before the formal investigation, a pilot study was conducted among the consumers with experience of short video ads in six cities in China. The questionnaire was revised in response to the feedback to ensure the content validity of the scale. Then a formal structured questionnaire was designed on WJX, a professional platform working on questionnaire design and data collection. Thanks to the assistance of marketing research association of Chinese universities, the questionnaire data were collected by the council members of the association in Beijing, Shanghai, Guangdong, Shaanxi, Sichuan, Jiangsu, and other provinces in China. The survey lasted for 1 month and collected a total of 420 questionnaires, of which 41 questionnaires with inconsistent answers or those finished in <2 min were taken as invalid and thus eliminated, and 379 questionnaires were found valid for the final analysis, with an effective recovery rate of 90%.

The demographic characteristics of the subjects are listed in Table 2. Age characteristics show that the young consumers are in the majority; those between 18 and 25 years of age are accounting for the highest proportion (63%). The gender characteristics reflect almost equal proportion between male and female, in spite of a slightly higher proportion of female by 2.12%. As to education characteristics, the majority of consumers have higher education qualifications, in which bachelor's degree makes up the highest percentage (57%). In addition, in the characteristics of online shopping times, the largest ratio goes to those whose online shopping times are <6. Finally, the characteristics of monthly income reveal that the low-income group is in large number; those with an income of <3,000 yuan are accounting for the highest proportion (56%).

**Table 2:** Sample characteristics.

**Characteristics**	**Category**	**Percentage**	**Characteristics**	**Category**	**Percentage**
Age	0–18	2%	Gender	Male	48.94%
	18–25	63%		Female	51.06%
	26–30	10%	Number of online purchase	0–3	48%
	31–40	19%		4–6	32%
	40+	6%		7–10	9%
Education background	Master degree or above	37%		10+	11%
	Bachelor degree	57%	Monthly	¥0–3,000	56%
	Associate degree	4%		¥3,000–6,000	13%
	High school degree or less	2%		6,000-10,000	18%
				¥10,000	13%
Total					100%

#### Measurement of Variables

To ensure the reliability and validity of measurement, this study employed the scale derived from the relevant concept of authority and 7-point Likert scale to measure the variables involved. First, to fully depict the concept of presence, we adapted phrasing in the preexisting scales from Klein, Bulu, Hassanein, and Head (Vijayasarathy, 2002; Klein, 2003; Hassanein and Head, 2007). Social presence, co-presence, and physical presence include three items, individually. Second, customer inspiration, in accordance with two-state 10-item scale of customer inspiration by Böttger et al. (2017) can be categorized into the following two dimensions: Cognitive activation and customer intention, namely, the dimension of “inspired-by” and of “inspired-to,” each of which contains five items. Third, impulsive purchase intention was measured by a rephrased scale derived from the research of Vijayasarathy (2002); three items involved in the measurement. Furthermore, all translated scales in the questionnaire were back-translated to validate the accuracy of phrasing in each item.

### Reliability and Validity Test

#### Reliability Test

In this study, the reliability of the variables was tested by Cronbach's α coefficient with a threshold of >0.7. The corrected item-to-total correlation coefficients (CITC) were calculated between each item and global item score with statistical significance set at >0.5, as suggested by Churchill. The reliability values of each variable are listed in Table 3.

**Table 3:** Results of reliability analysis.

**Variable**	**Indicator**	**Cronbach′s α **	**CITC**	**Cronbach′s α **
				**if item deleted**
Social presence	sp1	0.878	0.759	0.832
	sp2		0.776	0.817
	sp3		0.758	0.832
Co-presence	cp1	0.845	0.703	0.792
	cp2		0.759	0.737
	cp3		0.674	0.821
Physical presence	pp1	0.878	0.779	0.815
	pp2		0.821	0.778
	pp3		0.698	0.886
Customer inspiration	ci1	0.937	0.742	0.930
	ci2		0.680	0.933
	ci3		0.770	0.929
	ci4		0.725	0.931
	ci5		0.642	0.935
	ci6		0.745	0.930
	ci7		0.786	0.928
	ci8		0.802	0.927
	ci9		0.773	0.929
	ci10		0.790	0.928
Impulse purchase intention	ibm1	0.911	0.705	0.900
	ibm2		0.749	0.895
	ibm3		0.717	0.898
	ibm4		0.691	0.901
	ibm5		0.763	0.893
	ibm6		0.755	0.894
	ibm7		0.726	0.897

It can be seen from the reliability analysis that the *Cronbach*′*s α* coefficients of all variables in the model are >0.8, and the CITC are >0.7, fully satisfying the criteria that *Cronbach*′*s α* is >0.7 and CITC is >0.5, suggesting that the reliability of the questionnaire and the internal structure consistency of variables are high.

#### Validity Test

Validity analysis is to test the rationality and validity of the questionnaire. The high validity level indicates that the measurement results are in good agreement with the measurement targets. In this study, the rationality and validity of the questionnaire were tested through construct validity (including convergent validity and discriminant validity) that refers to the extent of consistency between the theory and the data. Convergent validity is to assess whether the data of items in one dimension point to the same variable and correlations between different items are high. The discriminant validity refers to the assumption that the items having no correlation with the variables do have no correlation with the variables. Furthermore, the factor analysis was conducted in this study to evaluate the construct validity of the questionnaire.

The Kaiser–Meyer–Olkin (KMO) measure of variables and the Barlett test of sphericity are required before the factor analysis. The KMO statistic is used to test the partial correlation between the variables. Ranging between 0 and 1, a KMO value >0.9 is considered adequate for the factor analysis. On the other hand, Bartlett test of sphericity is used to assess the independence between the variables. The significance of probability (*p*) is used to judge the adequacy for factor analysis. The results of KMO measure and Bartlett test of sphericity of each variable are listed in Table 4.

**Table 4:** Results of KMO measure and bartlett test of sphericity.

	**KMO value**	**KMO criterion**	***P*-value in**
			**Bartlett Test (sig)**
Physical presence	0.715	0.7	0.000
Social presence	0.743		0.000
Co-presence	0.716		0.000
Customer inspiration	0.915		0.000
Impulse purchase intention	0.897		0.000

It can be seen that the KMO values of all variables are >0.7, and the probability *p*-values of χ^2^ in Bartlett test of sphericity achieve the criteria of significance, indicating that the factor analysis can be performed.

#### Convergent Validity Test

In this study, confirmatory factor analysis (CFA) was used to test the convergent validity of the model. The results of the measurement model are listed in Table 5. The standard deviation (STD) scores of all latent variables are >0.7; the composite reliability (CR) scores are higher than 0.8; and the scores of average variance extracted (AVE) are >0.5. Therefore, the reported results fulfill the criteria of convergent validity (STD > 0.5; CR > 0.6; AVE > 0.5); thus, suggesting a good convergence validity of the tested model.

**Table 5:** Convergence validity test of measurement model.

**Variable**	**Indicator**	**Estimate**	**S.E**.	**C.R**.	***P***	**STD**	**CR**	**AVE**
Social presence	sp1	1				0.832	0.878	0.7063163
	sp2	0.979	0.065	15.144	***	0.858		
	sp3	0.968	0.065	14.834	***	0.831		
Co-presence	cp1	1				0.788	0.845	0.650988
	cp2	1.152	0.09	12.86	***	0.884		
	cp3	0.998	0.082	12.098	***	0.742		
Physical presence	pp1	1				0.858	0.878	0.7154763
	pp2	1.049	0.062	16.805	***	0.928		
	pp3	0.856	0.061	13.966	***	0.741		
Customer inspiration	ci1	1				0.791	0.937	0.6780494
	ci2	0.961	0.081	12.445	***	0.796		
	ci3	0.926	0.084	12.99	***	0.796		
	ci4	0.964	0.078	11.746	***	0.719		
	ci5	0.933	0.075	11.886	***	0.737		
	ci6	0.992	0.078	10.179	***	0.748		
	ci7	1.238	0.092	13.389	***	0.881		
	ci8	1.261	0.092	13.774	***	0.915		
	ci9	1.285	0.095	13.54	***	0.9		
	ci10	1.329	0.096	13.8	***	0.919		
Impulse purchase intention	ibm1	1				0.734	0.911	0.5935749
	ibm2	1.001	0.079	12.659	***	0.781		
	ibm3	0.974	0.081	12.068	***	0.751		
	ibm4	0.99	0.085	11.686	***	0.719		
	ibm5	1.191	0.092	12.926	***	0.811		
	ibm6	1.108	0.087	12.782	***	0.81		
	ibm7	1.047	0.085	12.337	***	0.782		

***Significant when p <0.001.

#### Discriminant Validity Test

In this study, the discriminant validity was tested using the method of AVE, in which when the square root of AVE ($\sqrt{AVE}$) obtained from the variables is greater than the correlation coefficient between the variables, the scale has good discriminant validity. It can be seen from Table 6 that the correlation coefficients between the variables are <0.5 while $\sqrt{AVE}$ of each variable is greater than the correlation coefficient of other variables, which meets the requirements of the discriminant validity test, indicating a good discriminant validity of the scale.

**Table 6:** Discriminant validity test between variables.

**Variable**	**Social presence**	**Co-presence**	**Physical presence**	**Customer**	**Impulse**
				**inspiration**	**purchase**
					**intention**
Social presence	**0.8404**				
Co-presence	0.479***	**0.8068**			
Physical presence	0.470***	0.421***	**0.8459**		
Customer inspiration	0.264***	0.293***	0.454***	**0.8234**	
Impulse purchase intention	0.333***	0.406***	0.316***	0.336***	**0.7704**

***Significant when p <0.001, the values on the diagonal in bold are $\sqrt{AVE}$, and the values in lower triangle are Pearson correlation coefficients between the variables.

### Model Validation Analysis

#### Main Effect Test and Analysis

##### Main Effect Test

We used generalized least squares (GLS) method to test the mediating effect by constructing the structural equation model. Model 1, which includes exogenous (co-presence, social presence, and physical presence) and endogenous variables (impulse purchase intention), is to verify Hypotheses H1a, H1b, and H1c. The results are listed in Table 7, that social presence, co-presence and physical presence have significant impact on impulse purchase intention. Therefore, H1a, H1b and H1c are all supported.

**Table 7:** Results of main effect calculation and goodness of fit.

**Model**	**Variable**		**Estimate**	**S.E**.	**C.R**.	**Hypothesis**	**CMIN/**	**NFI**	**RFI**	**IFI**	**CFI**	**RMSEA**
	**Exogenous**	**Endogenous**					**DF**					
M1	Social presence	Impulse purchase intention	0.330***	0.079	3.939	H1a	2.706	0.912	0.893	0.943	0.943	0.080
	Co-presence	Impulse purchase intention	0.373***	0.117	3.332	H1b						
	Physical presence	Impulse purchase intention	0.241**	0.112	2.820	H1c						

**Indicates significance level P <0.01, ***Indicates significance level P <0.001.

##### Main Effect Analysis

It can be seen from the results that the social presence has a significant positive impact on impulse buying intention, which supports Hypothesis H1a and is consistent with conclusions from the prior studies that social presence directly promotes impulse buying (Jiang et al., 2014). Contrary to the findings of the recent researches, social presence in the form of short video marketing does not result in the information degradation caused by the information overload. The information overload describes the state when information deteriorates because of overabundance of information source (Lin and Liu, 2007; Roetzel, 2019). Increasing the number of redundant information diminishes the marginal line between important and unimportant information when excessive stimuli occur, which might lead to the degradation of information in form of mediocrity (Lin and Liu, 2007; Roetzel, 2019); in other words, the previously important information becomes less important. Comparatively, in spite of the instant exposure of a large amount of visual and auditory information to short video consumers, the information related to social presence plays the same significant role as it does in the traditional web marketing (Jiang et al., 2014)—a direct impact on impulse purchase intention, as shown in the results of this study.

Similarly, the results also suggest that Hypothesis H1b holds true as co-presence has a significant positive impact on impulse buying intention. There are two levels in co-presence as follows: One level is to feel the virtual existence of others, that is, to perceive others; the other level is to feel that others are actively perceiving us, or accepting us being part of a group (Slater, 1999). This implies that when consumers perceive being part of the consumer group with others in the virtual world, their impulsive purchase intention may be easily triggered. Therefore, it can be inferred from the light of the conclusion of Hypotheses H1a and H1b that presence of human and social elements (Shi et al., 2021) may be a crucial driver of the consumption impulse in online shopping.

In addition, the findings also support Hypothesis H1c since the direct effect of physical presence on impulsive purchase is significant. However, compared with the findings in Hypotheses H1a and H1b, the direct effect of physical presence on impulsive purchase is significantly smaller than that of social presence and co-presence, which implies that social factors (social presence and co-presence) may produce stronger impact on impulsive purchase than physical factors (physical presence). It also confirms the findings in the prior studies that the most obvious inferiority of e-commerce to traditional face-to-face business is the absence of human feeling (Shi et al., 2021) and interaction (Gefen and Straub, 1997), anonymity and automation (van der Heijden, 2003; Wang and Emurian, 2003), which, to some extent, may impede the online transaction. For these reasons, the approaches to the enhancement of presence in managerial practice such as dynamic graph and 3D display fail to “make a product in vogue” or “create a sales myth” instantly as short videos have done. Another verified conclusion by the findings, as already shown in the previous work, is that physical presence, co-presence, and social presence do not always correlate with each other (Slater et al., 1999; Rui and Wang, 2008).

#### Mediating Effect Test and Analysis

##### Mediating Effect Test

Models 2–4 are constructed to test Hypotheses H2a, H2b, and H2c. Model 2 includes social presence (exogenous variable), customer inspiration (mediating variable), and impulse purchase intention (endogenous variable); Model 3 includes co-presence (exogenous variable), customer inspiration (mediating variable), and impulse purchase intention (endogenous variable); and Model 4 physical presence (exogenous variable), customer inspiration (mediating variable), and impulse purchase intention (endogenous variable). Bootstrapping technique and product of coefficients methods were employed to calculate the mediating effect. The results are listed in Table 8. Specifically, the non-standard indirect effect of Model 2 shows that 0 < lower < upper (0 <0.243 <0.509) and *z* ≥ 1.96, which indicates the existence of the mediating effect; thus, Hypothesis H2a holds. Similarly, in model 3, the non-standard indirect effect shows 0 < lower < upper (0 <0.260 <0.586) and *z* ≥ 1.96, indicating a mediating effect, and Hypothesis H2b holds. Finally, the non-standard indirect effect of Model 4 shows 0 < lower < upper (0 <0.278 <0.579) and *z* ≥ 1.96, so the mediating effect exists and Hypothesis H2c proves true.

**Table 8:** Results of mediating effect.

**Model**	**Hypothesis**	**Variable**		**Effect**	**Estimates**	**Product of coefficients**		**Bootstrapping**				**Conclusion**
		**Exogenous**	**Endogenous**					**Bias-corrected** **95% CI**		**Percentile** **95% CI**		
						**SE**	**Z**	**Lower**	**Upper**	**Lower**	**Upper**	
M2	H2a	Social presence	Impulse purchase intention	Total effect	0.589	0.068	8.662	0.460	0.728	0.455	0.725	Social presence → **Customer inspiration**→
				Indirect effect	0.364	0.068	5.353	0.243	0.509	0.237	0.503	Impulse purchase intention **Mediating effect exists**
M3	H2b	Co-presence	Impulse purchase intention	Total effect	0.702	0.078	9.000	0.553	0.860	0.547	0.857	Co-presence → **Customer inspiration**→
				Indirect effect	0.410	0.084	4.881	0.260	0.586	0.257	0.583	Impulse purchase intention **Mediating effect exists**
M4	H2c	Physical presence	Impulse purchase intention	Total effect	0.557	0.069	8.072	0.418	0.693	0.423	0.700	Physical presence → **Customer inspiration**→
				Indirect effect	0.411	0.076	5.408	0.278	0.579	0.276	0.573	Impulse purchase intention **Mediating effect exists**

##### Mediating Effect Analysis

According to the results of this empirical study, inspiration plays a mediating role in the impact of physical presence, social presence, and co-presence on impulse buying, which confirms Hypotheses H2a, H2b, and H2c. As addressed by the previous studies, inspiration is in the position to change views and perceptions (Oleynick et al., 2014; Figgins et al., 2016), and plays a mediating role between antecedent variables and outcome variables (Thrash et al., 2010). Our results verify inspiration as a motivational state that brings ideas to fruition (Oleynick et al., 2014). The obtainment of physical presence, social presence, and co-presence allows consumers to be better informed of the product and imagine its influence on their off-line life (Rauschnabel et al., 2019), which may well lead to an inspiring moment of “Aha” that facilitates a rapid transition of behavior (Böttger et al., 2017). The above conclusion confirms customer inspiration as a key concept in the explanation of consumer behavior (Böttger et al., 2017; Rauschnabel et al., 2019), which is especially important for understanding impulsive purchase behavior in short video context.

Study 1 examines the influencing mechanism of presence on impulse purchase intention by a questionnaire survey. Next, an experimental research was conducted to further testify the robustness and universality of the findings in Study 1. One of the advantages of experimental method is to measure more accurately the two temporary psychological and intentional variables, customer inspiration, and impulse purchase intention in short video scenario. On the other hand, the causal relationship between presence and impulse purchase intention can be validated. The experiment in this study consists of a pilot study and three experimental studies. The pilot study is to test whether the selected short video material would successfully stimulate presence of the subject. Studies 2, 3, and 4, respectively, examine the main effects of social presence and physical presence on impulse purchase intention, and the mediating effects of customer inspiration.

## Pilot Study: Pretest

### Manipulation Materials for Presence

A pretest was conducted in this study to verify the validity of presence manipulation in the experiment. As already known, social presence highlights the sense of communication and interaction with others in the media environment; co-presence refers to a sense of being together with others in the shared space (Horvath and Lombard, 2010); and physical presence addresses a sense of immersion and realism of the virtual environments (Bulu, 2012). Accordingly, two marketing experts and a team of graduate students worked together to script and shoot three groups of short videos, namely, Groups A, B, and C, respectively, to trigger social, physical, and co-presence. We selected fascia gun, hoodie, and office chair as the objects introduced in the three groups of videos. Each group had two videos (e.g., A1 and A2) which should maintain consistent in the introduction of properties of product such as appearance, material, and function, but different in stimulation of presence. For example, video A1 performed better than video A2 in terms of communication and interaction, that is, A1 could trigger high social presence while A2 could trigger low social presence. Similarly, video B1 generated higher sense of being together than video B2. So, videos B1 and B2 stimulated high and low co-presence, respectively. Since video C1 evoked more sense of immersiveness than video C2, high and low physical presence were stimulated, respectively.

### Manipulation Test

Sixty subjects were recruited on the Credamo platform, including 16 males and 44 females, who were randomly divided into two groups, and asked to watch videos A1 and A2. Afterward, they were required to fill out the questionnaire for social presence. Independent sample *t*-test shows that the social presence triggered by watching video A1 (M_highsocialpresence_ = 4.88, SD = 1.40) is significantly higher than that by video A2 [M_lowsocialpresence_ = 3.12, SD = 1.82; *t*(58) = 4.20, *p* = 0.000]. So, the manipulation on social presence was successful.

Likewise, another 60 subjects, 17 males and 43 females, were recruited on the Credamo platform and randomly divided into two groups. After watching videos B1 and B2, their co-presence was measured by the relevant questionnaire. Independent sample *t*-test indicates that the co-presence (M_highco−presence_ = 4.80, SD = 1.26) produced by watching video B1 is significantly higher than that produced by video B2 [M_lowco−presence_ = 4.02, SD = 1.21; *t*(58) = 2.43, *p* = 0.018]. Thus, the manipulation of co-presence was successful.

The same procedure was repeated by the researchers to recruit another 60 participants (26 males and 34 females) on the platform as the subjects of test on physical presence. Also, they were divided into two groups, and watched videos C1 and C2, respectively. Consequently, the independent sample *t*-test of the data from physical presence questionnaire shows that video C1 (M_highphysicalpresence_ = 5.12, SD = 1.40) triggers higher physical presence than video C2 does [M_lowphysicalpresence_ = 4.22, SD = 1.21; *t*(58) = 2.66, *p* = 0.010], so the physical presence was successfully manipulated.

## Study 2: Experiment on Social Presence

Study 2 aims to verify the positive impact of social presence on impulse purchase intention in short video context and the mediating effect of customer inspiration.

### Experimental Design

Videos in Group A in the pretest were selected as the short video material in Study 2. Based on the experimental procedure suggested by Rook and Fisher (1995), a single-factor experiment on social presence (high *vs*. low) was designed as follows:

*Experimental situation: You are going to buy a pair of socks online*.

*Financial condition: Currently your spare money for living is 500 yuan, and still have 5 days to go before the 2000 yuan for living expenses next month added to your account*.

*Shopping discovery: You are to buy socks introduced in a short video before you happen to see another video about a fascia gun, a kind of vibration massage device to relax muscles. You have been longing to have such a gun, and the present discount price for sales is 299 yuan*.

The fascia guns in video A1 and video A2 were exactly the same in properties such as appearance, material, and price, but the anchor showed better communication skills in A1 than in A2. Therefore, A1 tended to motivate high social presence, while A2 low social presence. The financial constraints were designated in this scenario to highlight the conflict so that the seduced buying behavior in case of a shortage in cash can be confidently identified as impulsive purchase.

### Experimental Procedure

**Figure 2:**
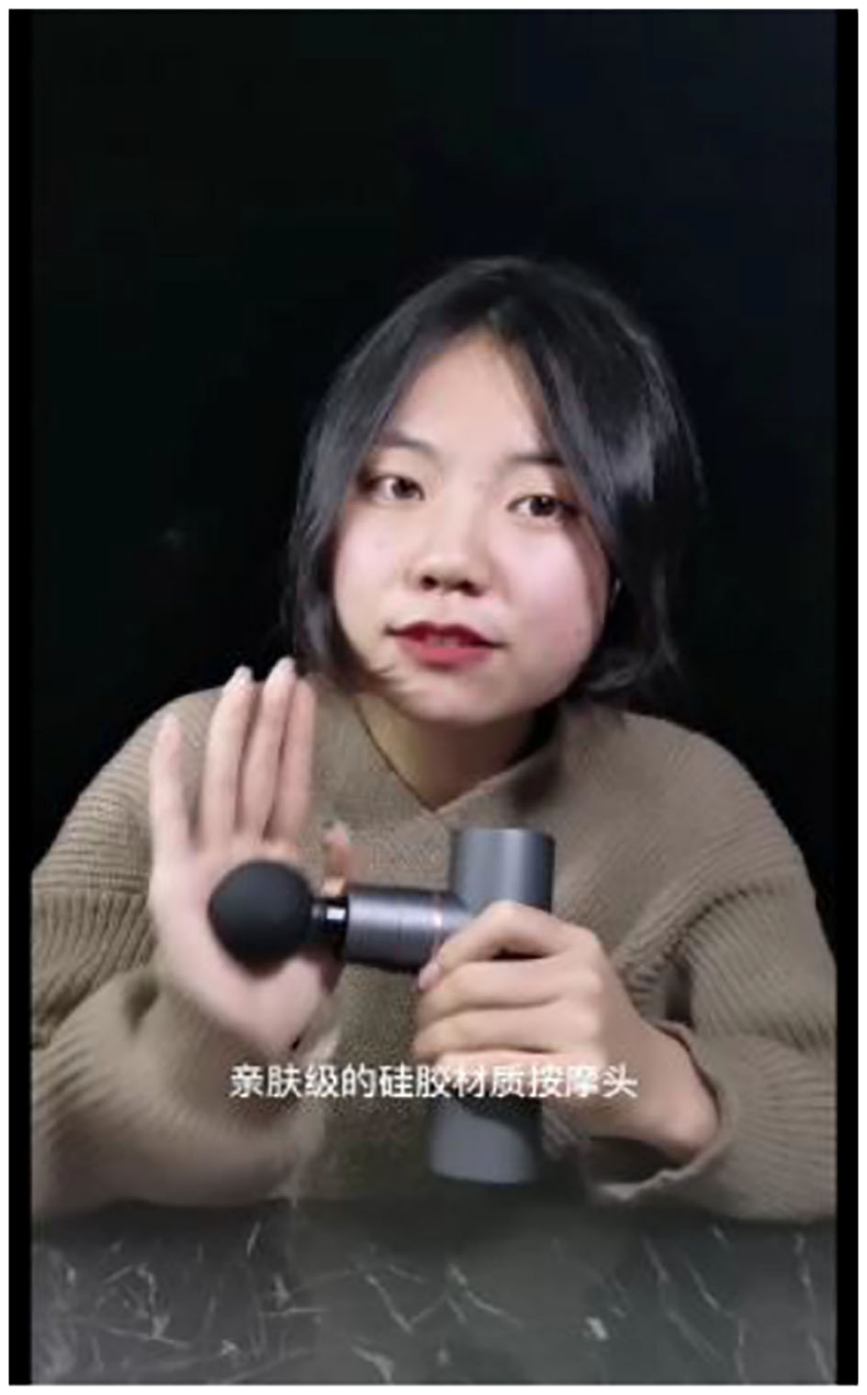
A1 Screenshot of high social presence.

**Figure 3:**
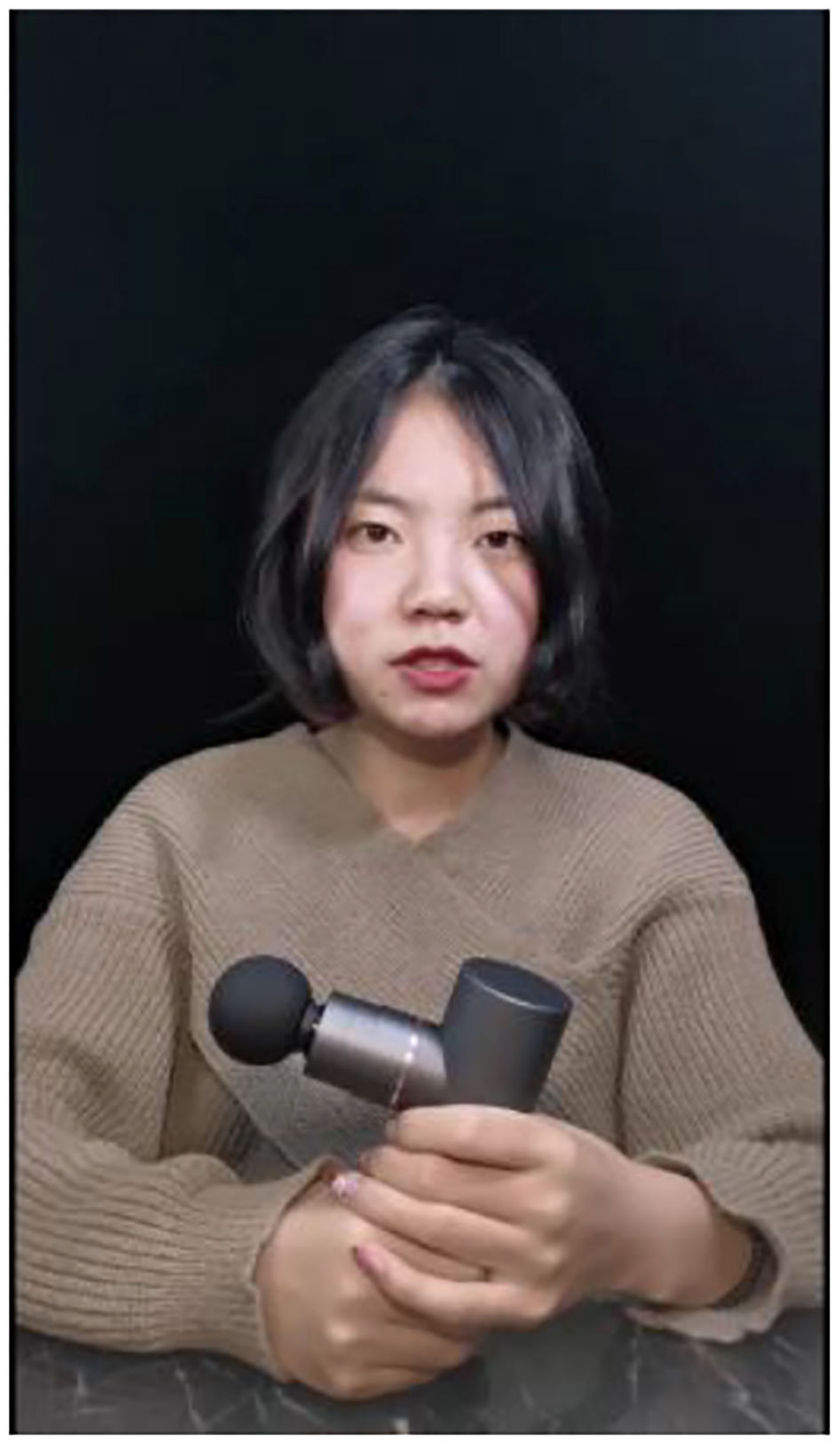
A2 Screenshot of low social presence.

A total of 150 participants (62 males and 88 females) recruited on the Credamo were randomly arranged to watch videos A1 and A2 after reading the condition described in Study 2 (screenshots of videos A1 and A2 are shown in Figures 2, 3). Then the researchers measured their social presence, perceived customer inspiration, and impulse purchase intention. The measurement items on impulse purchase intention were adapted from Yong et al. (2013): “While watching the video, even if the fascia gun is not in my shopping list, I have an impulsive buying desire, I want to buy it, and I am inclined to buy it.” Other items in the questionnaire were kept consistent with the previous ones in this study. Additionally, the participants needed to answer a question to see whether they have watched the entire video so that the eligible questionnaires could be sorted out. Finally, questions concerning demographic information including gender, age, education, the average monthly frequency of short video shopping were answered by the participants.

### Experimental Results Analysis

#### Social Presence Manipulation Test

We first tested whether videos A1 and A2 successfully stimulate social presence of the subjects. It is shown by a one-way analysis of variance (ANOVA) that social presence triggered by video A1 (M_highsocialpresence_ = 4.65, SD = 1.31) is significantly higher than that by video A2 [M_lowsocialpresence_ = 3.45, SD = 1.57; *F*_(1, 148)_ = 26.410, *p* = 0.000]. The significant difference indicates a success of manipulation on social presence.

#### Main Effect Test

**Figure 4:**
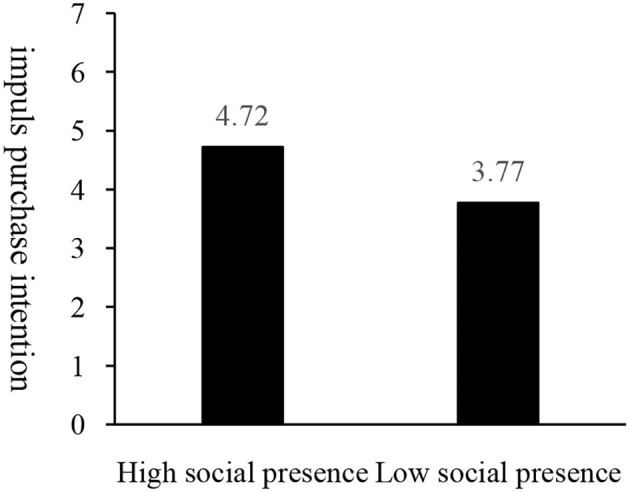
Impact of social presence on impulse purchase intention.

The ANOVA results show a significant higher impulse purchase intention in the group with high social presence (M_highsocialpresence_ = 4.72, SD = 1.57) than that with low social presence [M_lowsocialpresence_ = 3.77, SD = 1.64; *F*_(1, 148)_ = 13.264, *p* = 0.000] as shown in Figure 4, indicating a significant main effect of social presence on impulse purchase intention, thus Hypothesis H1a being verified.

#### Mediating Effect Test

A one-way ANOVA with customer inspiration as dependent variable indicates that customer inspiration of the high social presence group (M_highsocialpresence_ = 4.83, SD = 1.29) is significantly higher than that of the low social presence group [M_lowsocialpresence_ = 4.02, SD = 1.45; *F*_(1, 148)_ = 13.057, *p* = 0.000]. Then the mediating effect of customer inspiration was tested by Bootstrapping. In specific, Bootstrap procedure was performed with 5,000 samples on model 4 in which social presence was assessed as independent variable, impulse purchase intention as dependent variable, and the customer inspiration as mediating variable. The results show that the mediating effect of customer inspiration is significant (0.6428) when the 95% confidence interval does not contain 0 (LLCI = 0.4925, ULCI = 0.8020), which verifies Hypothesis H2a that the customer inspiration plays a mediating role in the impact of social presence on impulse purchase.

## Study 3: Experiment on Co-presence

Study 3 aims to verify the positive impact of co-presence on impulse purchase intention in short video context and the mediating effect of customer inspiration.

### Experimental Design

Videos in Group B in the pretest were used as the materials in Study 3. A single-factor experiment on co-presence (high vs. low) was constructed.

*Experimental situation: You are going to buy a pair of socks online*.

*Financial condition: Currently your spare money for living is 500 yuan, and still have 5 days to go before the 2000 yuan for living expenses next month added to your account*.

*Shopping discovery: You are to buy a pair of socks introduced in a short video before you happen to see another video about a hoodie that is really a kind of your favorites, and the present discount price for sales is 256 yuan*.

The experimental design was identical to that of Study 2. In spite of the consistency of the basic properties of hoodies in videos B1 and B2, the short video anchor showed a better sense of togetherness in video B1 than in video B2. That is, video B1 stimulated high level of co-presence while B2 low level.

### Experimental Procedure

**Figure 5:**
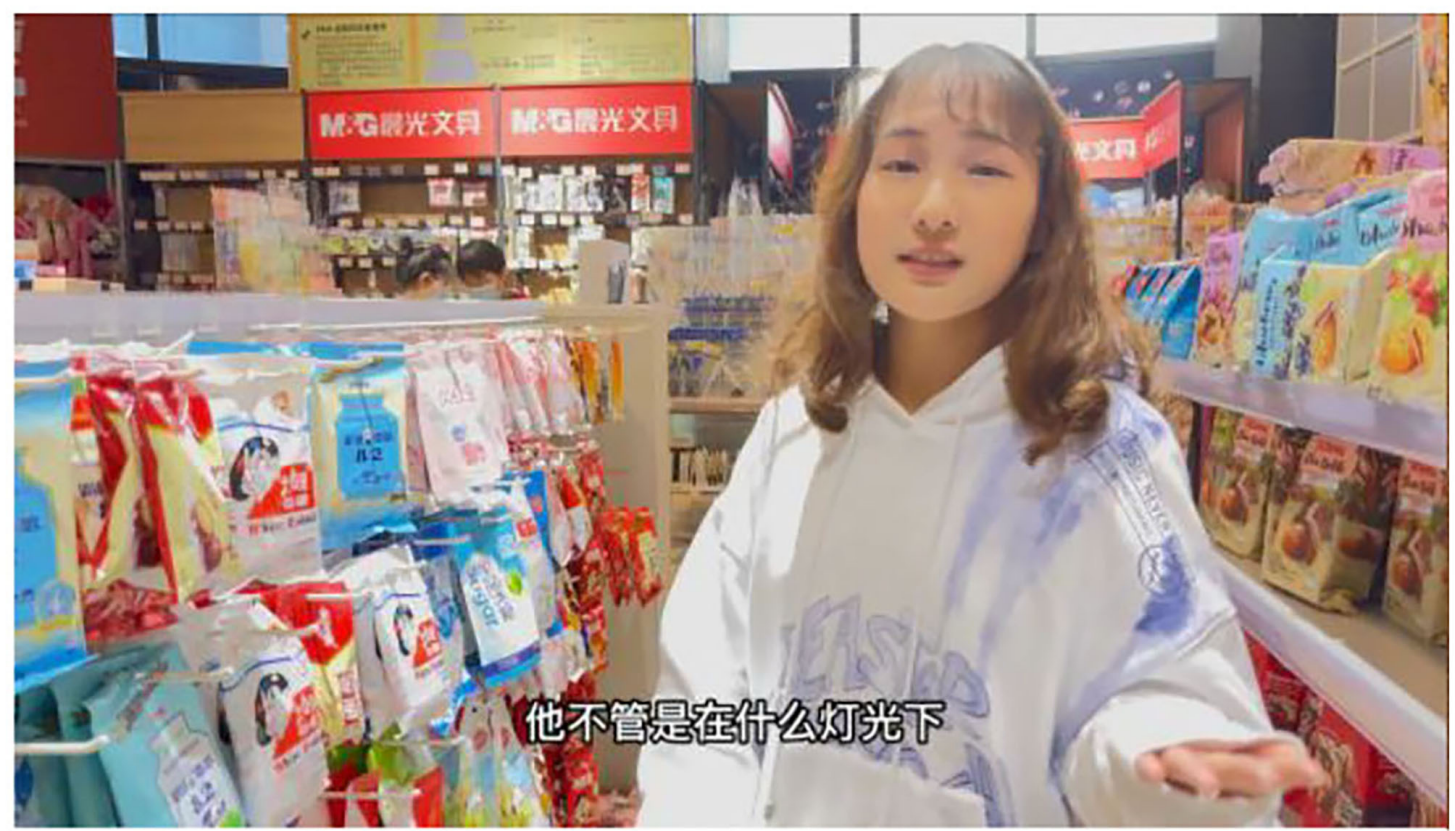
B1 Screenshot of high co-presence.

**Figure 6:**
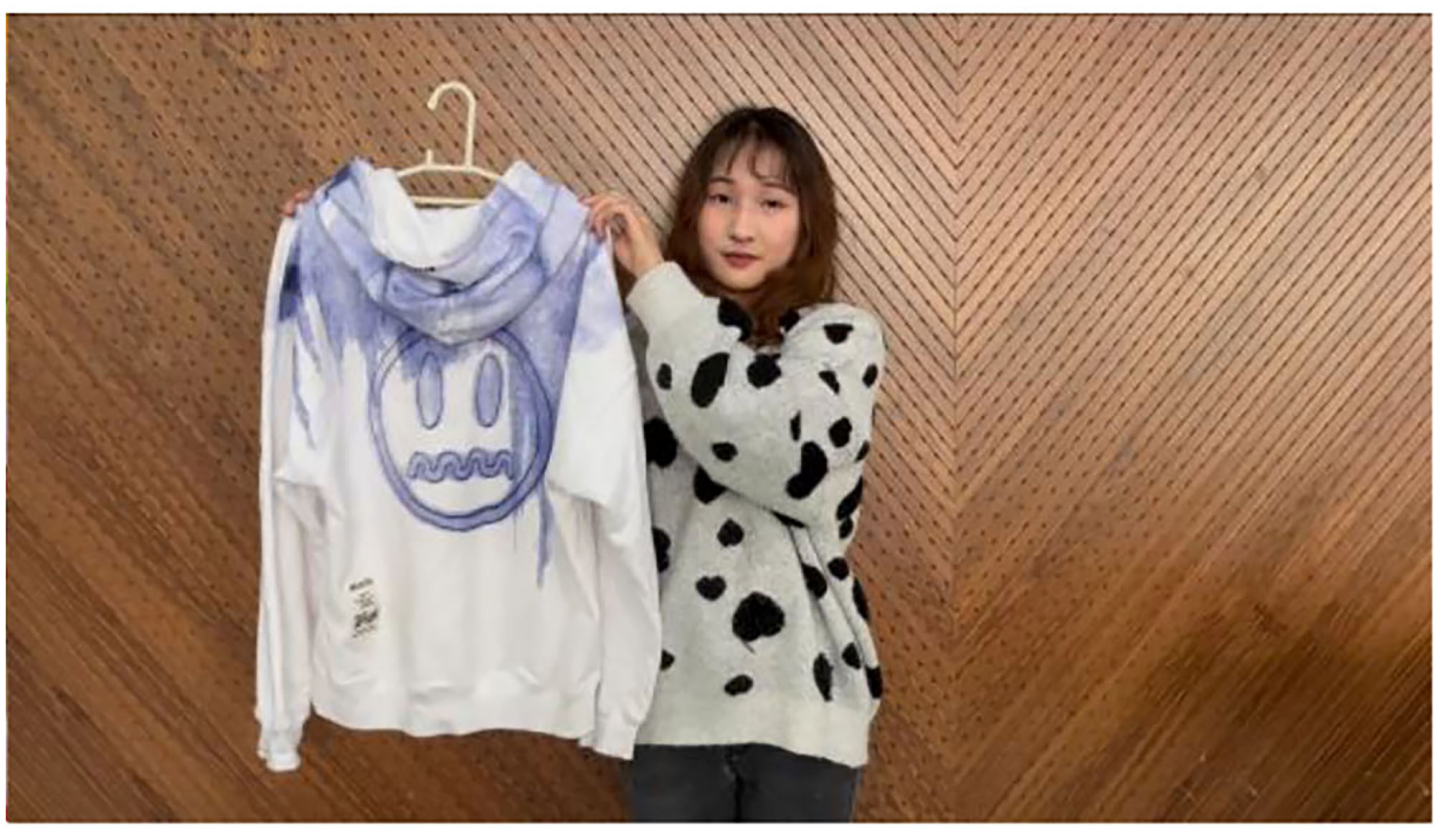
B2 Screenshot of low co-presence.

A total of 150 participants were recruited on the Credamo platform, including 61 males and 89 females. After reading the experimental condition in Study 3, they were randomly exposed to videos B1 or B2 (screenshots of videos B1 and B2 are shown in Figures 5, 6). The participants' impulse purchase intention, as well as their perceived co-presence and customer inspiration were measured sequentially afterward. All measurement items were the same as in Study 2. Similarly, a question about the short videos watched in Study 3 and questions about the demographic information of the subjects were also included in the questionnaire.

### Experimental Results Analysis

#### Co-presence Manipulation Test

We firstly tested whether videos B1 and B2 successfully inspire the subjects' co-presence. A one-way ANOVA shows that the level of co-presence stimulated by video B1 (M_highco−presence_ = 5.23, SD = 1.01) is significantly higher than that by video B2 [M_lowco−presence_ = 3.40, SD = 1.44; *F*_(1, 148)_ = 49.85, *p* = 0.000], indicating that the manipulation on co-presence was successful.

#### Main Effect Test

**Figure 7:**
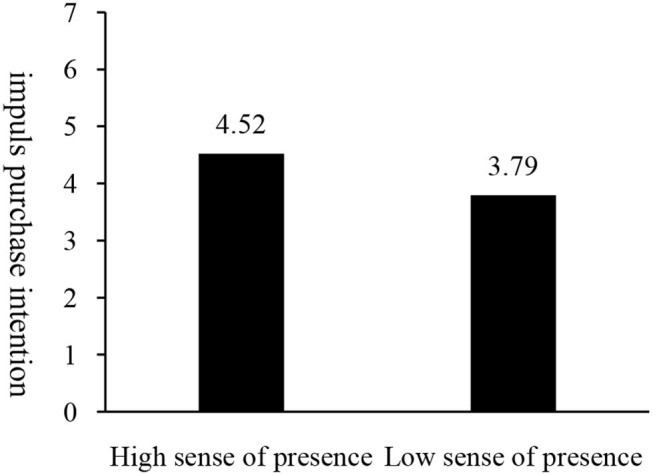
Impact of co-presence on impulse purchase intention.

ANOVA indicates that the impulse purchase intention (M_highco−presence_ = 4.52, SD = 1.48) in high co-presence group is significantly higher than that in the low co-presence group (M_lowco−presence_ = 3.79, SD = 1.54; *F*_(1, 148)_ = 8.71, *p* = 0.004). As shown in Figure 7, thus, the main effect of co-presence on impulse buying intention is significant, and Hypothesis H1b verified.

#### Mediating Effect Test

First, one-way ANOVA was performed with customer inspiration as the dependent variable. The result shows that the customer inspiration (M_highco−presence_ = 4.69, SD = 1.12) in the high co-presence group is significantly higher than that in the low co-presence group [M_lowco−presence_ = 4.02, SD = 1.38; *F*_(1, 148)_ = 10.72, *p* = 0.001]. The mediating effect of customer inspiration was then tested by Bootstrapping with 5,000 samples on model 4 considering co-presence as independent variable, impulse purchase intention as dependent variable, and customer inspiration as mediating variable. The result indicates that the effect of mediation was significant (0.6126) if the 95% confidence interval does not contain 0 (LLCI = 0.4446, ULCI = 0.7884), which again verifies Hypothesis H2b, namely, customer inspiration acts a mediating role in the impact of co-presence on impulse purchase.

## Study 4: Experiment on Physical Presence

Study 4 aims to verify the main effect of physical presence on impulse purchase intention and the mediating effect of customer inspiration in short video context.

### Experimental Design and Procedure

**Figure 8:**
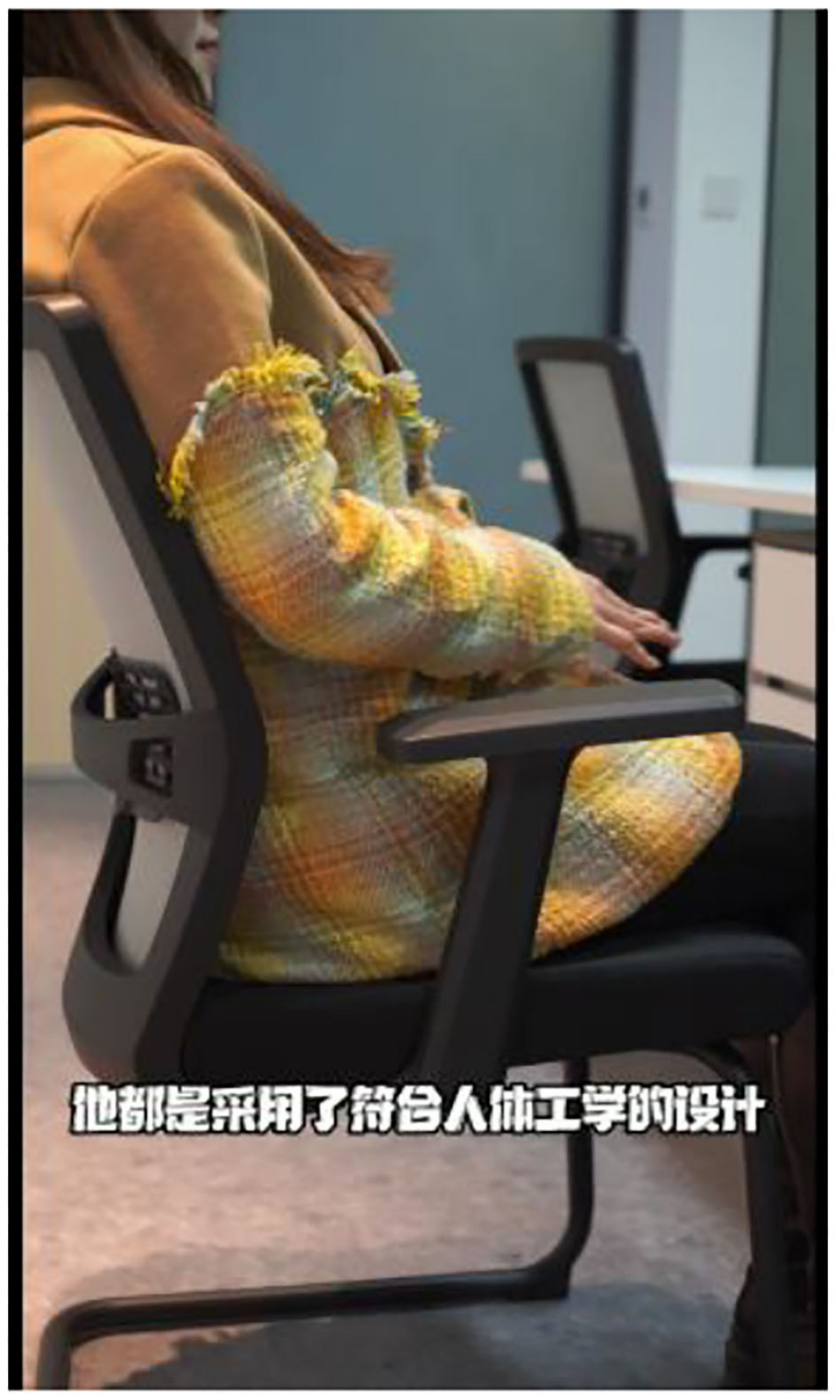
C1 Screenshot of high physical presence.

**Figure 9:**
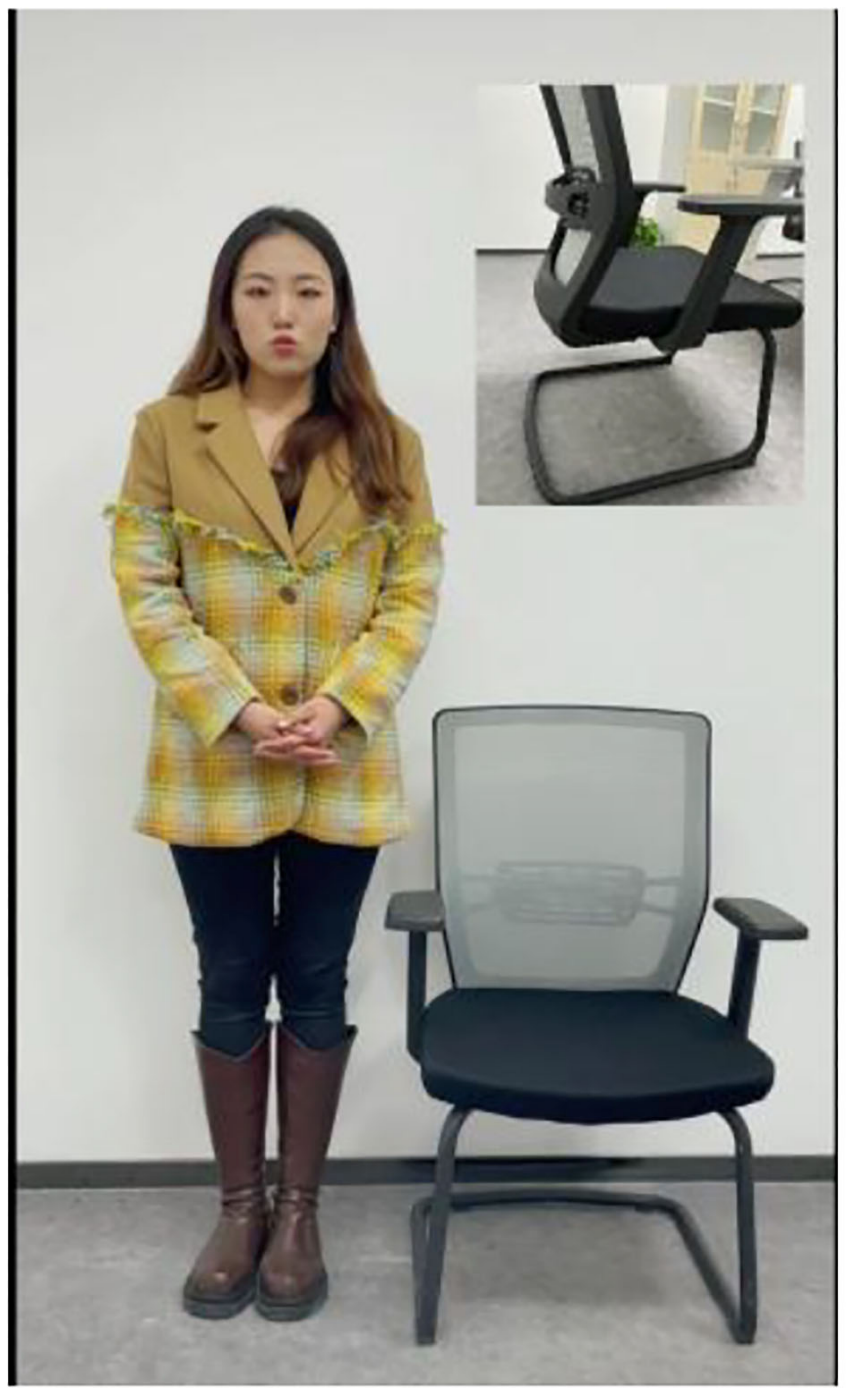
C2 Screenshot of low physical presence.

Videos in Group C in the pretest were used as the materials in Study 4, and a single-factor experiment on physical presence (high *vs*. low) was adopted. The features of the office chairs introduced in videos C1 and C2 maintained consistent, although video C1 could more effectively arouse the sense of immersiveness than video C2. Thus, it can be asserted that video C1 and C2 stimulated high and low physical presence, respectively. A total of 160 participants recruited on the Credamo platform, including 71 males and 89 females, watched the videos and answered the questionnaire identical to that in Study 3. Screenshots of videos C1 and C2 are shown in Figures 8, 9.

### Experimental Results Analysis

#### Physical Presence Manipulation Test

Likewise, stimulation test on physical presence was conducted on the subjects after they had watched videos C1 and C2. A one-way ANOVA shows that the physical presence inspired by video C1 (M_highphysicalpresence_ = 4.71, SD = 1.24) was significantly higher than that by video C2 [M_lowphysicalpresence_ = 4.12, SD = 1.27; *F*_(1, 158)_ = 8.78, *p* = 0.004]. Such a significant difference indicates a success of manipulation on physical presence.

#### Main Effect Test

**Figure 10:**
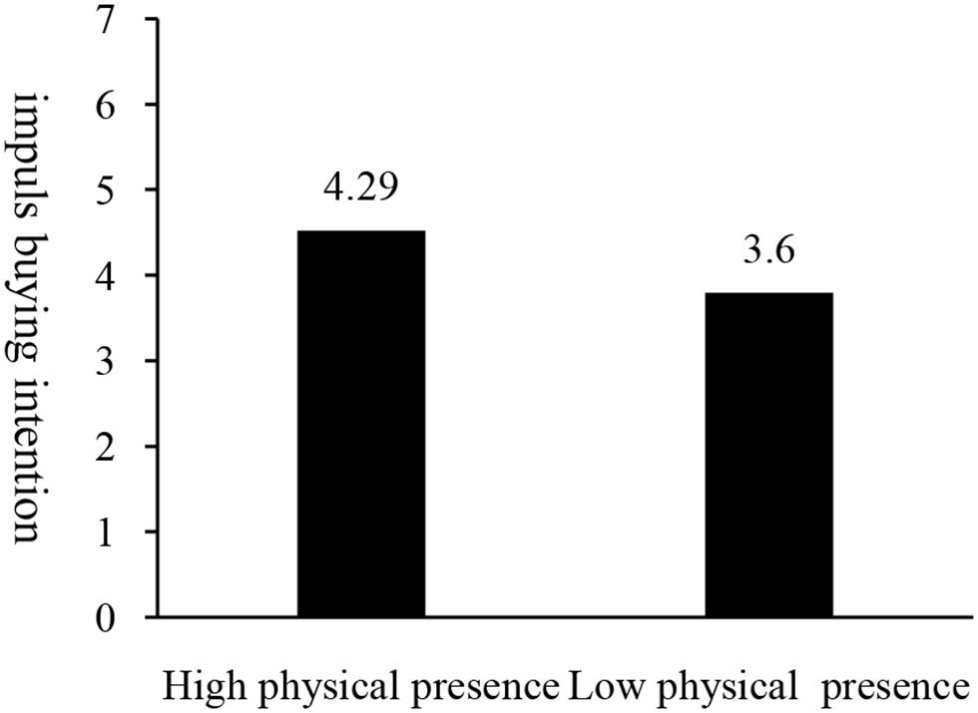
Impact of physical presence on impulse purchase intention.

The ANOVA test shows a higher intention of impulse purchase in the high physical presence group (M_highphysicalpresence_ = 4.29, SD = 1.66) than in the low physical presence group [M_lowphysicalpresence_ = 3.60, SD = 1.67; *F*_(1, 158)_ = 6.82, *p* = 0.010]. As shown in Figure 10, therefore, the main effect of physical presence is significant and Hypothesis H1c verified.

#### Mediating Effect Test

First, one-way ANOVA was conducted with customer inspiration as the dependent variable. It is shown that customer inspiration of the high physical presence group (M_highphysicalpresence_ = 4.53, SD = 1.40) is significantly higher than that of the low physical presence group [M_lowphysicalpresence_ = 3.91, SD = 1.52; *F*_(1, 158)_ = 7.04, *p* = 0.009]. Second, the mediating effect of customer inspiration was analyzed by Bootstrapping with 5,000 samples on model 4, taking physical presence as independent variable and impulse purchase intention as dependent variable. The result confirms a significant mediating effect (0.8653) when the 95% confidence interval does not contain 0 (LLCI = 0.7348, ULCI = 1.005), which verifies Hypothesis H2c that customer inspiration plays a mediating role in the impact of physical presence on impulse buying.

## Conclusion and Implication

### Conclusion

This study focuses on the psychological mechanism of impulsive purchase in short video. Through a questionnaire survey and three situational experiments, the study tests the relationship among presence, short video customer inspiration, and impulse purchase intention. The empirical results show that the following:

(1) Social presence, co-presence, and physical presence have significant positive impacts on impulsive purchase intention.
(2) Customer inspiration plays a mediating role in the impact of physical, social, and co-presence on impulsive purchase.
(3) Social and co-presence have a stronger impact on impulse purchase intention than physical presence, which confirms that social factors produce stronger influence on impulsive purchase in short video environment than physical scenes.

### Implications for Theoretical Field

“Short video + e-commerce” has presented a comparatively better capacity to guide shopping and marketing than graphs and text in traditional e-business. However, the previous studies on consumer purchasing decision in short video context neither clearly explain why the purchase conversion rate of short video is much higher than that of graphic mode nor clearly explain the psychological mechanism of impulsive purchase. For these reasons, this study, based on the theory of presence—a core difference between short video and traditional graphic displaying, tends to explore from the perspective of customer inspiration the specific mechanism in which the extrinsically stimulated motivation can be transformed to an instant purchasing behavior. It contributes to the theoretical field in the following two aspects:

(1) By testing the direct effect of presence—namely, social presence, co-presence, physical presence—on impulse purchase in short videos, the study validates the effect of “presence,” a traditional e-commercial element, on consumer behavior in the booming short video marketing environment. The findings suggest that social presence, co-presence and physical presence have significantly positive impacts on impulse purchase intention, but the influence coefficient indicates social factors (co-presence and social presence) produce stronger impact on impulse purchase than situational factors (physical presence) in short video context, and thus supporting the prior studies in that the roles of physical presence, co-presence and social presence do not always correlate (Slater et al., 1999; Rui and Wang, 2008).

(2) This study depicts the mediating mechanism of customer inspiration between presence and customer impulse purchase in the context of short video marketing. It is apparent that short videos create a better user experience with rich and dimensional content than the traditional displaying methods such as text and graphs. The behavioral shift from being attracted to being impulsed to buy, however, requires psychological motivation. Customer inspiration involves the motivation to expand the qualities embodied in the summoned object (Thrash and Elliot, 2003). Therefore, this study innovatively introduces the concept of customer inspiration to analyze the impulse buying behavior of short video viewers. It is shown in the findings that customer inspiration mediates the impact of physical, social and co-presence on impulse purchase, which demonstrates the significance of customer inspiration in the short customer journey from “being inspired by” to “being inspired to” under short video marketing scenario. It confirms that inspiration, as noted in many studies, is an emerging and promising key construct in marketing research that can be applied to further interpretation of consumer behavior (Böttger et al., 2017; Rauschnabel et al., 2019).

### Implications for Marketing Practice

In short video e-commercial mode, consumers are allowed to place their orders in the purchase page while they are watching the short video. Such an “check out” mode successfully facilitates the transition from customer inspiration evoked by sense of presence in short videos to a temporary buying behavior. The short video ads can be very effective to this transition especially when they are perceived with a higher degree of involvement and immersion. The official statistics from Taobao revealed a 20% higher purchase conversion rate by short videos than the traditional graphic mode (Dong, 2019), and the annual advertising revenue of TikTok was estimated to amount to 20 billion yuan in 2018.

#### Implications for Consumers

Since social presence, co-presence and physical presence produce significant positive impacts on impulsive purchase intention, as indicated by the results, consumers who are watching short videos are suggested to raise their purchase rationality and make reasonable decision in line with their actual needs and purchasing power, which is especially true when there is such high interactivity between short video anchor and audiences as conversational or responsive communication, explosion of likes or comments, and other similar situations.

#### Implications for Short Video Anchors and Brand Owners

It is suggested to increase presence of consumers by enhancing their viewing experience with smooth pictures and lifelike features, so that the time for decision-making can be effectively reduced and the purchase conversion rate thus improved. More precisely, the enhancement of physical, social, and co-presence in short video will elicit customer inspiration that can be converted into buying behavior over a short period of time. Therefore, technologies such as virtual and augmented reality (Rauschnabel et al., 2019), digital signage, and online tools (Böttger et al., 2017) could be used in the production of short videos to create quasi-reality experience for consumers and thus increase physical presence. Similarly, social elements could be inserted to short videos through immersive visual content and interactive expressions to increase social and co-presence that can inform customers with better knowledge about the product, enrich their imagination on its practical uses, stimulate customer inspiration, and facilitate actual conversion from an idea to purchasing behavior.

## Limitations and Future Research

In spite of the contributions, this study has several limitations. First, this study explores the effects of short video (short video presence) on customer inspiration and impulsive purchase intention based on the previous theories on inspiration and characteristics of short video. Unfortunately, despite the antecedents of customer inspiration, other possible drivers of customer inspiration deserve further research that would possibly bring new insight to short video marketing. Second, the this study mainly focuses on the behavioral outcomes from customer inspiration, but lays little emphasis on the impact of customer inspiration on emotion and attitude. Future research could investigate the relationship between customer inspiration and existing marketing constructs (e.g., the effect of customer inspiration on satisfaction and loyalty). Third, this study does not explore the boundary conditions for customer inspiration in short video. Although the prior studies once suggested the moderating effect of inspiration recipient's traits on customer inspiration, it is still not clear what and how the specific traits of the recipient will moderate inspiration in short video context.

## Data Availability Statement

The raw data supporting the conclusions of this article will be made available by the authors, without undue reservation.

## Ethics Statement

Written informed consent was obtained from the individual(s) for the publication of any potentially identifiable images or data included in this article.

## Author Contributions

PG: conceptualization, methodology, writing—review and editing, supervision, funding acquisition, and writing—original draft. YZ: investigation and resources. YC: data curation and visualization. All authors contributed to the article and approved the submitted version.

## Funding

This work was supported by grant from the National Natural Science Foundation of China (71972156, 71802158), Shaanxi Social Science Fund (2018S42), Shaanxi Natural Science Fund (2014JM9370), and Special Research Projects of Shaanxi Provincial Department of Education (18JK0760).

## Conflict of Interest

The authors declare that the research was conducted in the absence of any commercial or financial relationships that could be construed as a potential conflict ofinterest.

## Publisher's Note

All claims expressed in this article are solely those of the authors and do not necessarily represent those of their affiliated organizations, or those of the publisher, the editors and the reviewers. Any product that may be evaluated in this article, or claim that may be made by its manufacturer, is not guaranteed or endorsed by the publisher.
